# The impact of social welfare support on mental health in Vietnam

**DOI:** 10.1371/journal.pone.0318374

**Published:** 2025-04-09

**Authors:** Duc Hong Vo, Tam Luong Huynh, Chi Minh Ho, Quynh Tran-Truc Vo

**Affiliations:** Research Centre in Business, Economics & Resources, Ho Chi Minh City Open University, Ho Chi Minh City, Vietnam; University of Georgia, UNITED STATES OF AMERICA

## Abstract

This study examines the impact of government support on mental health in Vietnam using Vietnam’s Households Living Standard Surveys in 2018 and 2020 and a probit estimator. Characteristics of the households and the households’ heads are also examined. We find that government support tends to worsen mental health in Vietnam, implying the current Government support is insufficient to improve mental health in households, particularly during stressful times during the COVID-19 pandemic. Female-headed households appear to experience a more significant mental health deterioration compared to their counterparts, whereas households living in urban areas are mentally struggling compared to those living in rural areas. Our results also indicate that mental health deterioration exhibits an inverted U-shaped relationship with age, implying mental health appears to be a significant issue for young individuals in Vietnam. Household incomes and assets act as a buffer against mental health deterioration. These findings support the view that mental health deterioration appears to emerge from financial distress. Households suffer mental health deterioration if their financial circumstances are not improved and support from the government is insufficient.

## 1. Introduction

“*Health is a state of complete physical, mental, and social well-being and not merely the absence of disease or infirmity*.” [1]

Mental health is one of the crucial factors contributing to overall health in every aspect of human life. Studies on mental health have largely been neglected due to a lack of mental health literacy, social stigma, cultural and social features, and discrimination in many countries, including Vietnam. Several societies, including Vietnam, have critical perceptions of individuals who are suffering from mental health issues. These perceptions contribute to the prejudices toward mental illness. More seriously, they form a barrier to discourage people from getting support to overcome their mental problems [2,3]. However, mental health has been gradually recognized, discussed, and gained more attention from policymakers and the public. Mental health has been qualitatively discussed in selected studies in the Vietnamese context [4–7]. A meeting was conducted in Hanoi in October 2022 with the theme “Making Mental Health and Well-being for All a Global Priority” to celebrate World Mental Health Day, raise awareness about mental health worldwide and encourage support for those struggling with mental health problems.

However, these positive signals do not mean that mental health is no longer a significant problem in modern society. A report by Vietnam’s Ministry of Health Ministry indicates that 14.9 per cent of Vietnamese (approximately 15 million people) suffer from one of the ten popular mental disorders. While anxiety and depression are believed to affect 5–6 per cent of the population, schizophrenia affects 0.47 per cent of the total population. The remaining health problems include bipolar affective disorder and drug- and alcohol-related psychosis. The Vietnamese government reported that around 15 per cent of the population required mental health care services in 2018. The COVID-19 pandemic has deteriorated mental health issues, particularly in emerging markets, and Vietnam is no exception. The pandemic ruined every aspect of human life, from the economies to the societies and, importantly, the healthcare system.

The International Labour Organization [8] reports that the pandemic caused the loss of jobs and endangered the livelihood of millions of people. Millions of women’s and men’s food security and nutrition were at risk when the breadwinner lost their jobs, got sick and passed away. Those who live in low-income countries, especially the most economically disadvantaged, for example, small-scale farmers, were hurt severely. Regarding the psychological aspect, there have been detrimental psychological consequences such as anxiety, depression, and many others in the societies due to lockdown policies, social distancing, quarantine, online homeschooling, the closing of businesses and loss of jobs [9–11]. Furthermore, data collected for this research also illustrates the sizable number of people dealing with mental problems. Table 1 indicates that approximately 14 per cent of people interviewed suffered mental health issues during the pandemic.

**Table 1:** The percentage of interviewees suffering mental health deterioration (MHD).

	Observations		Percentage	
	*2018*	*2020*	*2018*	*2020*
With MHD	6,212	1,273	13.55%	13.56%
Without MHD	39,627	8,116	86.45%	86.44%
Total	45,839	9,389	100%	100%

In the US, to lessen the pandemic’s detrimental impact on households’ financial situations, Congress created the American Rescue Plan Act, which included a temporary enlargement of the Child Tax Credit. Although much research showed that the 2021 CTC reduced childhood poverty and improved material well-being [12,13], credit had no impact on the parents’ mental health [14]. However [15], found that families with low earnings can lower anxiety and depression compared to those having higher earnings, and those without children, thanks to CTC 2021.

In Vietnam, efforts have been made focusing on mental health and government support. For instance [16], assessed the frequency and severity of anxiety and depression symptomatology, access obstacles to mental health services, and correlations between functional impairment and cancer inpatient status by using a cross-sectional study recruited adult cancer patients. Accordingly, 46.3 per cent and 27 per cent of people surveyed report depression and anxiety symptoms, respectively. Also, in the context of the COVID-19 pandemic [10,17,18], provided an insight into the mental health issues of healthcare professionals who were participating in preventing the spread of COVID-19 in Ho Chi Minh City, Vietnam. Besides, government support such as cash transfers reduces poverty and increases school enrolment in Vietnam [19]. Regardless of these efforts, the effect of government support on mental health does not attract much attention in Vietnam, particularly during the COVID-19 pandemic. On the one hand, government support is a major channel for improving social wellbeing. On the other hand, individual mental health is a critical factor in social well-being. A study [20] has recently confirmed the effect of cash transfers on improving individual well-being in a province in Vietnam. However, the authors have only treated mental health as an element of individual well-being. Therefore, there is an urgent need for academic studies to examine directly the impact of government support on mental health in the Vietnamese context, especially after the COVID-19 pandemic.

Mental health policies are designed to ease patients’ financial burdens when seeking treatment. However, the mental health care network in Vietnam is still limited in quantity and quality. Thirty-seven provinces and cities in Vietnam do not have clinical psychologists. More than 11,000 commune health stations only provide free medicine for people with schizophrenia, epilepsy, and depression. Other services such as screening, therapy, relapse prevention or rehabilitation are unavailable. The proportion of psychiatric clinics in the private sector is also relatively small [21,22]. Moreover, many fiscal policies were also enacted to ease the burden on people during the hardship caused by COVID-19. As such, it is unclear if this type of policy can help lessen the mental problems related to mental health that the residents have faced.

Against this backdrop, this study is one of the first to examine the effects of government support on mental health faced by Vietnamese households. We particularly focus on the effect of government support on mental health, incorporating the characteristics of the household heads, such as gender, age, and educational level. Our analysis also examines the role of household finance, such as income and assets, in mental health in Vietnam before and during the COVID-19 pandemic.

Following this introduction, the remainder of this paper is structured as follows. Section 2 provides a literature review on relevant issues in the existing literature. Section 3 describes the research methodology and data. Section 4 presents and discusses the empirical results regarding the effects of the government support policies on Vietnamese mental health, followed by the concluding remarks and policy implications in section 5 of the paper.

## 2. Literature review

A large body of literature examines the effect of government support on people’s welfare. However, examining its impact on mental health is still limited in the existing literature. Mental health has recently gained significant attention from governments, practitioners, and the public. Cheng et al. 2021 examined the mental health of UK working parents during the COVID-19 pandemic. The results reveal that deteriorating mental health conditions have become more severe for working parents and are closely associated with significant financial instability, caring time for children, and homeschooling. As such, regardless of income level for the households, men or women, the deterioration in mental health has become unbearable. [23] found that mental health issues among males have been getting worse. Age, religion, educational attainment, and financial stability of the family are significant determinants of mental health issues.

Besides, government aid programs also have significant impacts on mental health. Numerous studies have included government support as a key independent variable [24–27]. Some studies have used government support as the moderating factor in the government mental health nexus. Zimmerman et al. [25] considered that cash transfers, commonly referred to as direct payments made to people living in poverty, may positively affect the mental health of young people. Chatterjiet al. [26] showed that social support and government aid were associated with better mental health among women living in a rural area in Maharashtra, India. Evidence from Thailand and Vietnam also confirmed that benefits from fiscal policy, particularly government financial support programs, are associated with happiness and improved mental health in households during the COVID-19 pandemic [27].

Government support is also used as a moderating factor in examining its effect on mental health during hardship. For example [28], stated that aggressive labour market programs and social welfare services could mitigate the negative impacts of the economic crisis on people’s mental health. [29] suggested that financial assistance from the government lessens the depression and anxiety symptoms linked to the lockdowns. Economic assistance is considered a good intervention which can reduce the negative effect on people’s mental health. Additionally, countries with social safety nets experience smaller changes in mental health related to economic downturns. Findings from empirical studies conducted in Finland and Sweden indicate that health inequalities have largely remained unchanged, and the suicide rates decreased during the economic recession with a significant rise in unemployment. Social benefits and services persisted and served as a buffer against structural pressures that would have led to wider health disparities [30–32].

On the one hand, academic papers confirm the positive effect of government support on people’s mental health. Still [33], provided mixed evidence because poverty alleviation programs such as cash transfers could positively and negatively impact mental health. [34] findings reveal that government aid only mitigates the psychological symptoms in non-urban areas. However, government aid alone is insufficient to address the population’s mental health. Findings from their study also reveal that government support may negatively affect mental health.

The existing literature has identified research gaps. Previous studies have used limited government support [25,33]. Government support in various forms, such as direct cash transfers, unemployment benefits, and mental health programs, should be considered to assess their relative impact and effectiveness on mental health for different populations. In addition, the impact of government support on mental health pre- and during the COVID-19 pandemic has not been thoroughly investigated.

Our literature review indicates that the effect of government support on mental health has largely been ignored in the Vietnamese context. As such, this study is warranted. We utilize the VHLSS surveys from two waves in 2018 and 2020—the latest surveys available when this study is implemented—to examine the effect of government support on the mental health of Vietnamese households.

## 3. Data and methodology

### 3.1. Data

This paper uses data from Vietnam Household Living Standard Surveys 2018 and 2020 (VHLSS2018 and VHLSS2020) conducted by Vietnam’s General Statistics Office (GSO). Since 2002, the GSO has conducted surveys every two years to monitor Vietnam’s population, supervise and evaluate the implementation of the comprehensive strategy on economic growth and poverty reduction, and comment on the assessment of the implementation results of the millennium development goals and socio-economic development goals of Vietnam.

### 3.2. Measuring mental health deterioration

Mental health deterioration is the process in which mental health becomes extremely worse over time. Normally, mental health deterioration can be identified by symptoms including schizophrenia, bipolar disorder, major depressive disorder, and many other severe symptoms such as mania, aggressive behaviour, and suicidal thoughts and behaviour. However, information from the VHLSS surveys does not provide any of the above information. As such, we develop a measure of mental health deterioration in Vietnam based on available responses from the questionnaires used in the surveys.

First, we consider people struggling with their lives with the responses to whether their living conditions have improved. Respondents with the response “I feel remain unchanged” or “my living conditions get worse” are recorded as those suffering from mental health deterioration. Second, we then follow the responses to the follow-up five questions used in the surveys. These questions include: (i) “Has any member of your family been sick or passed away?”; (ii) “Do you have to pay for food and other higher-priced consumer goods?”; (iii) “Do you earn low income?”; (iv) “Do you lose your job?”; and (v) “Is there any conflict between your family and others/neighbours?”. Answering “yes” for 3 out of these five follow-up questions, these respondents are identified as those suffering mental health deterioration. Our method is based on the procedure’s validation developed by [35,36]. These authors considered that respondents discussing the experience regarding losing a loved one, higher payment for consumer goods, low income, losing a job, and poor neighbour relationships are exposed to trauma and experience a deterioration in their mental health deterioration.

### 3.3. An analytical framework

The dependent variable, “mental health deterioration,” is binary. This variable is assigned the value of “1” for those experiencing mental health deterioration and “0” otherwise. In addition, the main independent variable used in our study is “government support”. Information regarding government support is collected from responses to the following questions in the VHLSS surveys, including: (i) “Did your household receive support from the supporting project?” and if yes “, How much did you receive?”. The government support is proxied by the total amount of money the households receive from the government.

**Fig 1:**
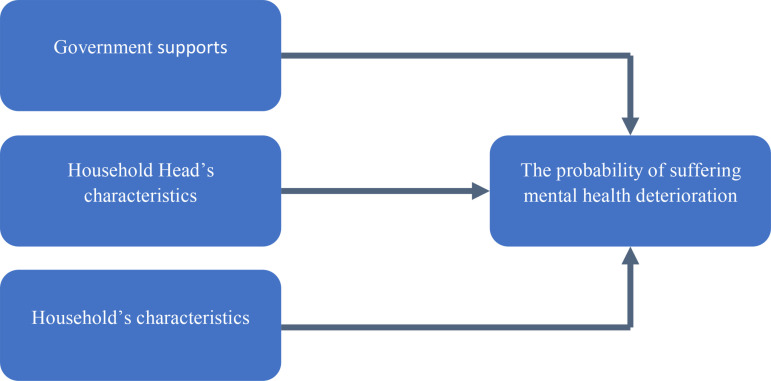
An analytical framework to examine the effect of government support on the probability of suffering mental health deterioration.

A set of control variables has been used in this study, including variables from (i) individual characteristics and (ii) household characteristics. For individual characteristics, gender equity is considered first. Gender equity has been widely discussed. [37] considered that the prevention targeted US women and the older ones who were widowed because they found significantly greater depressive disorders in females. The difference in depressive symptoms due to sex differences is also supported in various studies [38]. As such, we examine whether females are more vulnerable regarding mental health in this study using gender as a dummy variable, with “1” representing females and “0” for males. Fig 1 exhibits our analytical framework.

**Fig 2:**
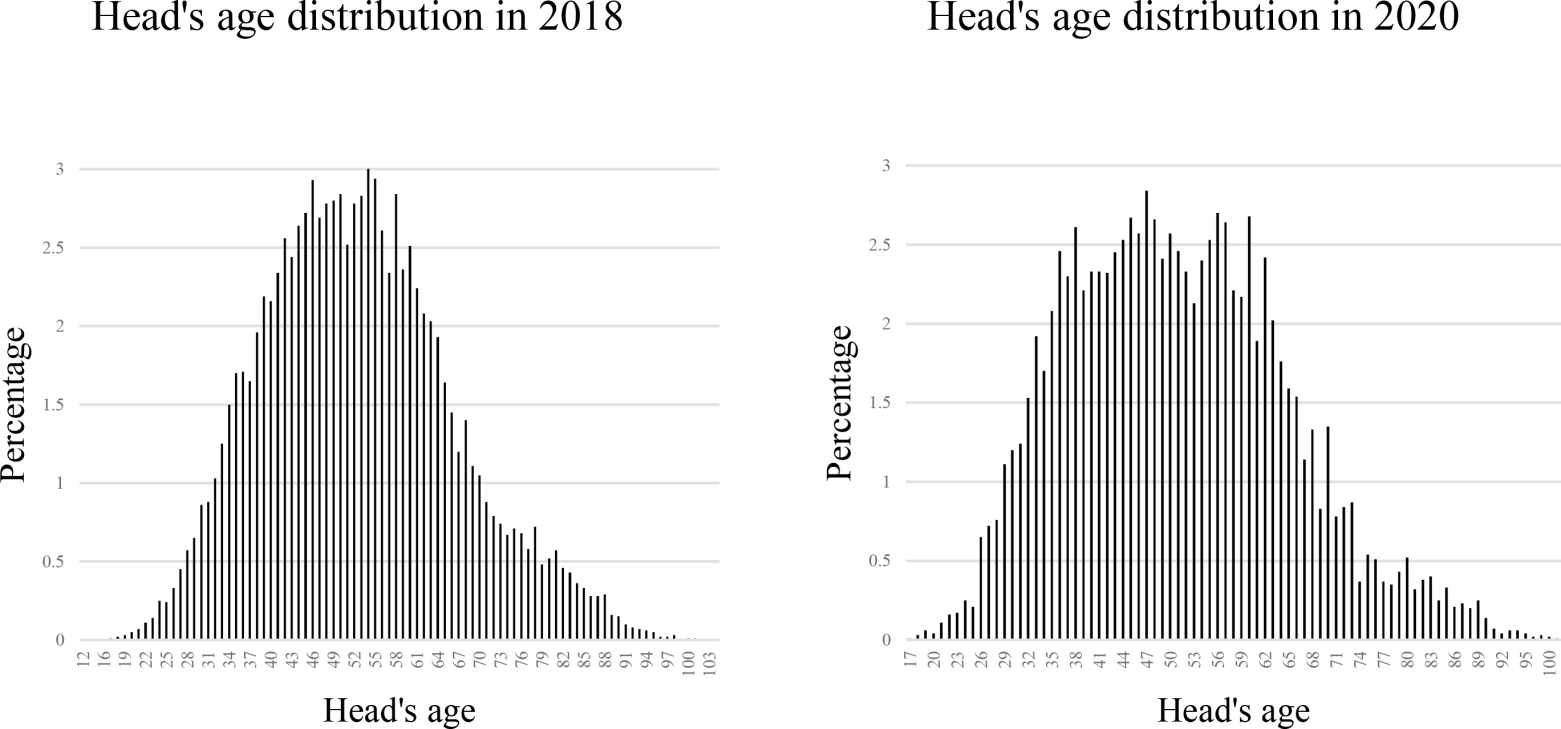
Age distribution of the interviewees from the VHLSS surveys in 2018 and 2020.

[39] considered that age, wage, income, asset, education level, and marital status are relevant. As such, these characteristics are also included in our analysis. Ageing is the main risk factor for many pathologies in human lives. Furthermore [40], also considered that those pathologies include mental health. Besides, a stereotype of older people is also relevant. Accordingly, older adults, such as disabled, weak, low, and helpless, can have a negative impact on their well-being. However, they hesitate to seek mental treatment due to the stigma. In the Vietnamese context, among the interviewees, people aged 45 to 55 account for a significant proportion (Fig 2). People in this age range are generally considered vulnerable and negatively impacted due to fast-paced living environments, social pressure, heavy workloads, and deterioration in physical function. Moreover, Vietnam is one of the fastest ageing population countries, implying that the number of older adults is growing. As a result, mental health deterioration has been alarming for these people. Besides [41], indicated a curvilinear relationship between age and well-being. As such, we incorporate this curvilinear relationship into our analysis using the square of the household member age.

Regarding education, the interviewees’ education levels are divided into five categories, including (i) having no diplomas, (ii) having pre-primary school diplomas, (iii) having secondary school diplomas, (iv) having high school diplomas, and (v) having graduate and post-graduate diplomas. Sociologists consider that marriage, in particular, and social connections, in general, can strengthen people’s connections to society and one another, thereby improving mental health [42]. Furthermore, different levels of mental health problems also happen in different living areas, such as urban and rural regions. This is because people living in cities have to confront loneliness, pollutants, and traffic congestion, which can have a severe psychological impact [43]. Like gender, living area is a dummy variable, taking the value of 1 if the person lives in an urban area and 0 otherwise. Finally, regarding the household’s characteristics, household size, which is calculated using the equivalent scale suggested by [44], is also included in our analysis. Finally, absent members in the interviewed household are identified as those who have not lived in that house for more than six months. Our regression is as follows:


$$MHD_{t}=\alpha +\beta_{1}logGOVS_{t}+\beta_{2}Householdhead^{\prime }scharacteristics_{t}$$

$$+\beta_{3}Household^{\prime }scharacteristics+\varepsilon_{i,t}$$ (1)

where **MHD** represents the mental health deterioration with the value of “0” being “no mental health deterioration” and a value of “1” being “having a mental health deterioration”; **GOVS** denotes the total monetary support from the governments; ***Household Head’s characteristics*** include gender, age, educational level, and marital status, head’s wage income; and ***Household characteristics*** include income (labour’s incomes, and incomes from doing agriculture, husbandry, agricultural services, forestry, aquaculture, and other income sources), household’s assets, whether the household living in the urban or rural areas, household size, and a number of absent members.

### 3.4. A research methodology

Our dependent variable is binary. As such, the conventional OLS estimation is not appropriate because the estimated coefficients from this OLS are biased and inefficient. As such, the Tobit and Probit estimation techniques are used in this analysis. However, the Tobit model is designed to estimate the linear relationship between variables when the dependent variable is left-censored, meaning it cannot take a value below a certain threshold, typically an equal threshold 0) or right-censored. As such, Tobit regression cannot be used in this analysis. On the other hand, probit regression is appropriate for use whenever modelling one of two alternatives occurs [45]. As such, probit estimation is used in this paper. However, the probit estimation only shows us the signs of the estimated coefficients regarding the correlation between government support and mental health deterioration in Vietnam. Their magnitudes are not reported. As a result, we also use the marginal effects to determine the probability of experiencing mental health problems when changes in government support emerge. We also conduct probit regression with provincial fixed effect and logit regression to confirm the robustness of our results. Results from fixed effect probit regression and logit regression are provided in the appendixes. Particularly, Appendix 1 to Appendix 4 exhibit the results from fixed effect probit regression, and the logit regression results are provided in Appendix 5 to Appendix 8. Appendix 9 presents the average marginal effect of the logit estimator on the probability that a household suffers from mental health deterioration for both rural and urban areas.

Table 2 summarises descriptive statistics for all variables used in our analysis. We observe the change in the government support between two surveys in 2018 and 2020 for pre-Covid-19 and during the Covid-19 period, respectively. The released data of VHLSS 2018 and 2020 show a notable difference in the number of observations. Even though the sample size is different between the two released surveys in 2018 and 2020, the randomness and representativeness of the data have remained the same, as affirmation by Vietnam’s General Statistics Office (GSO). The mental health deterioration distribution has no statistical difference between pre- and during COVID-19, as in 2018 and 2020. Notably, there is a difference in the median value of the government’s support between poor and non-poor households. Given the low proportion of poor households (7.12 per cent in 2018 and 7.55 per cent in 2020), the government’s support might be paid to a small fraction of the Vietnamese households in our sample. Additionally, the correlation matrix, included in Appendix 1, indicates no significant correlation between variables included in the empirical regression, with two exceptions. The correlation between male-headed and married households is approximately 0.6, and between married and widowed households is approximately −0.8.

**Table 2:** Summary of the descriptive statistics.

Variables	Mean		Median		Min		Max	
	*2018*	*2020*	*2018*	*2020*	*2018*	*2020*	*2018*	*2020*
***A full sample***								
MHD	0.131	0.136	0	0	0	0	1	1
GOVS (VND’000)	915.464	991.476	0	0	0	0	301,965	112,700
Head’s gender	0.748	0.736	1	1	0	0	1	1
Head’s age	52.396	50.981	52	50	12	17	113	101
Education diplomas								
+ Having no diploma	0.177	0.221	0	0	0	0	1	1
+ Pre-primary diploma	0.271	0.234	0	0	0	0	1	1
+ Secondary school diploma	0.310	0.287	0	0	0	0	1	1
+ High school diploma	0.161	0.173	0	0	0	0	1	1
+ Graduate and post-graduate diploma	0.081	0.085	0	0	0	0	1	1
Household head’s marital status								
+ Singled	0.028	0.322	0	0	0	0	1	1
+ Married	0.802	0.796	1	1	0	0	1	1
+ Widowed	0.135	0.130	0	0	0	0	1	1
+ Divorced	0.035	0.042	0	0	0	0	1	1
Head’s wage (VND’000)	72,596	91,500	47,510	66,000	0	0	1,462,000	1,013,700
Other incomes (VND’000)	135,018	164,191	35,457	58,100	0	0	43,200,000	52,600,000
Household asset (VND’000)	962,000	1,236,489	509,910	677,300	0	100	80,700,000	35,100,000
Households in urban areas	0.311	0.328	0	0	0	0	1	1
Household size (equivalent)	1.000	2.860	1	2.768	0.584	1	1	8.867
Number of absent members	0.119	0.104	0	0	0	0	5	4
Observations	34,358	9,389						
***Sub-sample of poor households***								
MHD	0.2829	0.2831	0	0	0	0	1	1
GOVS (VND’000)	5,042	6,164	2,550	2,772	0	0	161,193	112,700
Proportion of poor household	7.12%	7.55%						
***Sub-sample of non-poor households***								
MHD	0.119	0.124	0	0	0	0	1	1
GOVS (VND’000)	599.108	570.307	0	0	0	0	301,965	100,100
Proportion of non-poor household	92.88%	92.45%						

Note: **GOVS** denotes government support; **MHD** is mental health deterioration.

## 4. Empirical results and discussions

Table 3 presents the empirical results on the impact of government support on mental health deterioration in 2018 and 2020 using the average marginal effect from the probit estimator. *First*, our findings indicate that the probability of people suffering mental health deterioration is positively associated with government support. In other words, increases in government support are more likely to increase mental health deterioration in 2018 and 2020. This finding implies an insight that people who received support from the government feel that they need external support to fulfil their essential demands for daily life, while their neighbours do not. The negative feeling can build up an invisible pressure in their emotions and feelings about their living conditions. As such, even though government support can help improve urgent demand for several households, these supports create a barrier between receivers and non-receivers and might increase receivers’ negative feelings about their living conditions. Thus, support from the government might improve mental health deterioration in Vietnam. Our finding aligns with [34] finding, indicating that government support might deteriorate mental health. Income inequality between groups of individuals is evident, leading to the difference in living standards. The government will tax richer people’s income and redistribute tax revenue to the poor. As a result, high-income individuals may feel that government support negatively affects their motivation to work. Once the rich consider that their efforts and hard work are less rewarded due to high taxes and support benefiting others. As such, their ambition is lower, negatively affecting the quality of their mental health.

**Table 3:** The average marginal effect of the probit estimator of the probability that a household suffered from mental health deterioration in 2018 and 2020.

The dependent variable is the probability that a household suffers from mental health	2018	2020
GOVS (in log)	0.007***	0.008***
	(0.001)	(0.001)
Head’s gender is male	−0.017***	−0.026**
	(0.005)	(0.010)
Head’s age	0.004***	0.004***
	(0.001)	(0.002)
Square of head’s age	−0.025×10^−3^***	−0.024×10^−3^ *
	(0.000)	(0.000)
The household head’s education level is a		
Pre-primary diploma	−0.018***	−0.011
	(0.006)	(0.011)
Secondary school diploma	−0.054***	−0.045***
	(0.006)	(0.011)
High school diploma	−0.061***	−0.059***
	(0.007)	(0.012)
Graduate and post-graduate diploma	−0.098***	−0.093***
	(0.007)	(0.014)
Household head’s marital status b		
Married	−0.075***	−0.049**
	(0.013)	(0.023)
Widowed	−0.071***	−0.042 *
	(0.013)	(0.024)
Divorced	−0.016	0.014
	(0.016)	(0.029)
Head’s wage (in log)	−0.002***	−0.003***
	(0.000)	(0.001)
Other incomes (in log)	−0.005***	−0.006***
	(0.000)	(0.002)
Household asset (in log)	−0.021***	−0.014***
	(0.002)	(0.003)
Households in urban areas	0.064***	0.054***
	(0.005)	(0.009)
Household size (equivalent)	−0.262	−0.000
	(0.362)	(0.004)
Number of absent members	−0.001	0.025***
	(0.005)	(0.010)
Observations	34,358	9,364
Ward test ($\chi^{2}$ statistics)	1,596	405.55
	[0.000]	[0.000]
Probit model goodness-of-fit test ($\chi^{2}$ statistics)	34,546	9,380

Note: **GOVS** is Government supports; **MHD** stands for mental health deterioration;

(a ) reference category has no certificate;

(b ) reference category is single household’s head; Ward test ($\chi^{2}$ statistics) hypothesis is all coefficients are simultaneously equal zero; Probit model goodness-of-fit test ($\chi^{2}$ statistics) hypothesis is the model is not poor fitness; robust standard errors in parentheses; p-value of post estimation tests in squared brackets;

*** ,

** , and

*  are statistically significant at 1, 5, and 10 per cent, respectively.

*Second*, a negative relationship between the probability of mental health deterioration and male-headed households indicates that male is found to suffer mental health deterioration less than females. Women often earn less money compared to men because they are disadvantaged in access to the labour market. In addition, an inequality of job access between women and men exists in two surveys in 2018 and 2020. Females are loaded with family responsibilities, including raising children and caring, interrupting their career path or promotion at work, contributing to increased stress and mental health challenges. Moreover, women may face different social pressures and expectations, affecting their views and reliance on government support.

*Third*, the likelihood of having mental health problems is higher for people living in urban areas compared to those in rural regions. People living in the city are more likely to deal with mental health deterioration, possibly because urban environments are frequently fast-paced and high-stress. So, stress can be exacerbated easily by the demands of the job, high work intensity, noise, and many other factors.

Besides, an inverted U-shaped relationship between age and mental health deterioration is observed in both the 2018 and 2020 surveys. Mental health deterioration appears to increase to a certain age rather than decrease. Our empirical result also indicates that mental health deterioration follows a specific pattern over the lifespan. This result again affirms the finding of [41], confirming the nonlinear relationship between age and well-being. On the one hand, Vietnam faces an ageing population, as illustrated in Fig 2. On the other hand, the fast growth rate and the competitiveness characteristics of the emerging market in Vietnam put critical pressure on the labour force. Therefore, in early adulthood, impoverished individuals may face numerous challenges regarding securing stable employment, affordable housing, and meeting basic needs. These stress factors can contribute to poor mental health during this stage of life. However, the older people get, the more life experience and resilience they accumulate. They have faced and overcome various challenges and crises throughout their lives, which can equip them with enough experience to cope with the pandemic’s uncertainty and disruption, leading to improved mental health.

Our empirical results also indicate that individual characteristics such as education level and marital status significantly impact people’s mental health deterioration. However, the effect of these factors was more significant in 2018 than 2020 during the Covid-19 pandemic. Besides, household characteristics, including wage, income, and assets, decrease mental health. These findings support the view that financial buffer reduces mental health deterioration in Vietnamese households.

Table 4 presents the effect of government support on mental health deterioration between the advantaged (non-poor) and the disadvantaged (poor) groups of Vietnamese households. Given the possibility of a small proportion of households receiving government support (presented in Table 2), our results reveal that an increase in government support is positively associated with an increased probability of mental health deterioration in these two groups. The finding is in line with [34]. Especially the impact is higher for the poor because of poor physical health, crime, violence, or even stigma resulting from poverty, leading to increased mental health deterioration. This finding is also consistent with [46]. Additionally, the education level in a wealthy class of households significantly affected people’s mental health deterioration before the COVID-19 pandemic in 2018 and during the pandemic in 2020. However, the effect is less significant for the disadvantaged group of households. Our results indicate that when individuals acquire higher education qualifications, they are more likely to deal with mental health problems regardless of the crisis. People with a higher education level tend to earn more, leading to financial stability and a sense of security when they experience economic uncertainties caused by the pandemic. In addition, they are more aware of health care, especially mental health. These wealthy individuals quickly seek help and treatment for mental health problems. Our finding is consistent with Dalgard, Mykletun, Rognerud, Johansen, and Zahl’s (2007) findings. Notably, our findings also confirm the inverted U-shaped relationship between age and mental health deterioration for both groups of individuals.

**Table 4:** The average marginal effect of the probit estimator on the probability that a household suffers from mental health deterioration: the non-poor versus poor households.

The dependent variable is the probability that a household suffers from MHD	Non-poor households		Poor households	
	*2018*	*2020*	*2018*	*2020*
GOVS (in log)	0.003***	0.005***	0.009**	0.016**
	(0.001)	(0.001)	(0.005)	(0.006)
Head’s gender is male	−0.014***	−0.028***	−0.044	0.020
	(0.005)	(0.010)	(0.028)	(0.048)
Head’s age	0.004***	0.004**	0.015***	0.013**
	(0.001)	(0.002)	(0.003)	(0.006)
Square of head’s age	−0.021×10^-3^***	−0.019×10^-3^	−0.097×10^-3^***	−0.096×10^-3^ *
	(0.000)	(0.000)	(0.000030)	(0.000)
Household head’s education level				
Pre-primary diploma a	−0.021***	−0.005	0.035	−0.051
	(0.006)	(0.012)	(0.021)	(0.040)
Secondary school diploma a	−0.055***	−0.044***	−0.025	−0.035
	(0.006)	(0.011)	(0.023)	(0.048)
High school diploma, a	−0.062***	−0.057***	0.032	−0.019
	(0.007)	(0.012)	(0.038)	(0.078)
Graduate and post-graduate diploma a	−0.093***	−0.090***	−0.094	0.230
	(0.007)	(0.014)	(0.157)	(0.266)
Household head’s marital status				
Married b	−0.064***	−0.052**	−0.086 *	0.005
	(0.014)	(0.025)	(0.046)	(0.073)
Widowed b	−0.060***	−0.057**	−0.126***	0.076
	(0.014)	(0.026)	(0.045)	(0.075)
Divorced b	−0.011	−0.008	−0.029	0.206**
	(0.016)	(0.031)	(0.057)	(0.097)
Head’s wage (in log)	−0.002***	−0.003***	0.002	0.001
	(0.000)	(0.001)	(0.002)	(0.004)
Other incomes (in log)	−0.004***	−0.006***	−0.017***	−0.003
	(0.000)	(0.002)	(0.002)	(0.013)
Household asset (in log)	−0.020***	–0.012***	0.005	−0.019
	(0.002)	(0.003)	(0.008)	(0.016)
Households in urban areas	0.059***	0.053***	0.179***	0.065
	(0.005)	(0.009)	(0.034)	(0.055)
Household size (equivalent)	−0.255	0.003	0.000	−0.046**
	(0.344)	(0.004)	(0.000)	(0.020)
Number of absent members	−0.000	0.024**	−0.002	0.026
	(0.005)	(0.010)	(0.026)	(0.049)
Observations	31,912	8,659	2,446	705
Ward test ($\chi^{2}$ statistics)	1,178	289.06	245.94	49.85
	[0.000]	[0.000]	[0.000]	[0.000]
Probit model goodness-of-fit test ($\chi^{2}$ statistics)	31,963	8,661	2,426	705.71
	[0.390]	[0.437]	[0.512]	[0.302]

Note: **GOVS** is Government supports; **MHD** stands for mental health deterioration;

(a ) reference category has no certificate;

(b ) reference category is single household’s head; Ward test ($\chi^{2}$ statistics) hypothesis is all coefficients are simultaneously equal zero; Probit model goodness-of-fit test ($\chi^{2}$ statistics) hypothesis is the model is not poor fitness; robust standard errors in parentheses; p-value of post estimation tests in squared brackets; robust standard errors in parentheses;

*** ,

** , and

*  are statistically significant at 1, 5, and 10 per cent, respectively.

We also run the marginal effect of probit estimator in the sub-samples of female-headed households compared to male-head households and the sub-samples of urban and rural areas. The results are presented in Tables 5 and 6, respectively. Our results indicate that females experience mental illness more than men. Additionally, females living in urban areas are more likely to suffer mental health deterioration than those living in rural areas.

**Table 5:** The average marginal effect of the probability that a household is suffered from mental health deterioration: Female-headed households (FHHs) versus Male-headed households (MHHs).

The dependent variable is the probability that a household suffers from MHD.	*Sub-sample of FHHs*		*Sub-sample of MHHs*	
	*2018*	*2020*	*2018*	*2020*
GOVS (in log)	0.012***	0.010***	0.005***	0.008***
	(0.001)	(0.002)	(0.001)	(0.001)
Head’s age	0.006***	0.008***	0.003***	0.002
	(0.002)	(0.003)	(0.001)	(0.002)
Square of head’s age	−0.048 ×10^-^3***	−0.060 ×10^-^3**	−0.013	−0.000
	(0.000)	(0.000)	(0.000)	(0.000)
Household head’s education level				
Pre-primary diploma	−0.025**	−0.011	−0.017**	−0.012
	(0.012)	(0.023)	(0.007)	(0.013)
Secondary school diploma a	−0.057***	−0.053**	−0.054***	−0.043***
	(0.013)	(0.023)	(0.007)	(0.012)
High school diploma, a	−0.072***	−0.082***	−0.058***	−0.052***
	(0.015)	(0.026)	(0.008)	(0.014)
Graduate and post-graduate diploma a	−0.116***	−0.106***	−0.094***	−0.091***
	(0.016)	(0.032)	(0.008)	(0.015)
Household head’s marital status				
Married b	−0.072***	−0.056	−0.071***	−0.027
	(0.018)	(0.036)	(0.022)	(0.032)
Widowed b	−0.074***	−0.060 *	−0.064***	−0.019
	(0.018)	(0.036)	(0.024)	(0.039)
Divorced b	−0.055***	−0.053	0.039	0.118**
	(0.021)	(0.040)	(0.028)	(0.048)
Head’s wage (in log)	−0.005***	−0.004**	−0.001***	−0.002**
	(0.001)	(0.002)	(0.000)	(0.001)
Other incomes (in log)	−0.005***	−0.009***	−0.005***	−0.005***
	(0.001)	(0.003)	(0.000)	(0.002)
Household asset (in log)	−0.022***	−0.021***	−0.021***	−0.011***
	(0.003)	(0.006)	(0.002)	(0.003)
Households in urban areas	0.074***	0.032 *	0.059***	0.064***
	(0.009)	(0.017)	(0.005)	(0.010)
Household size (equivalent)	−0.687	0.003	0.000	0.000
	(0.478)	(0.009)	(0.000)	(0.004)
Number of absent members	−0.014	0.000	0.002	0.031***
	(0.013)	(0.026)	(0.005)	(0.010)
Observations	8,671	2,451	25,686	6,913
Ward test ($\chi^{2}$ statistics)	455.8	117.87	1,014	260.47
	[0.000]	[0.000]	[0.000]	[0.000]
Probit model goodness-of-fit test ($\chi^{2}$ statistics)	8,738	2,462	25,852	6,902
	[0.260]	[0.337]	[0.210]	[0.475]

Note: **GOVS** is Government supports; **MHD** stands for mental health deterioration;

(a ) reference category has no certificate;

(b ) reference category is single household’s head; Ward test ($\chi^{2}$ statistics) hypothesis is all coefficients are simultaneously equal zero; Probit model goodness-of-fit test ($\chi^{2}$ statistics) hypothesis is the model is not poor fitness; robust standard errors in parentheses; p-value of post estimation tests in squared brackets; robust standard errors in parentheses;

*** ,

** , and

*  are statistically significant at 1, 5, and 10 per cent, respectively.

**Table 6:** The average marginal effect for the probability that a household is suffered from mental health deterioration: Rural area versus Urban area.

The dependent variable is the probability that a household suffers from MHD.	*Sub-sample of urban area*		*Sub-sample of rural area*	
	*2018*	*2020*	*2018*	*2020*
GOVS (in log)	0.011***	0.008***	0.006***	0.007***
	(0.001)	(0.002)	(0.001)	(0.001)
Head’s gender is male	−0.011	−0.008	−0.022***	−0.035**
	(0.008)	(0.015)	(0.007)	(0.014)
Head’s age	0.006***	0.011***	0.004***	0.001
	(0.002)	(0.003)	(0.001)	(0.002)
Square of head’s age	−0.034×10^-3^**	−0.077×10^-3^***	−0.024×10^-3^***	0.000
	(0.000)	(0.000)	(0.000)	(0.000)
Household head’s education level				
Pre-primary diploma a	−0.026 *	−0.000	−0.014**	−0.016
	(0.013)	(0.023)	(0.006)	(0.012)
Secondary school diploma a	−0.069***	−0.024	−0.046***	−0.053***
	(0.013)	(0.022)	(0.006)	(0.012)
High school diploma, a	−0.065***	−0.047**	−0.059***	−0.066***
	(0.014)	(0.022)	(0.008)	(0.015)
Graduate and post-graduate diploma a	−0.107***	−0.093***	−0.098***	−0.086***
	(0.014)	(0.023)	(0.009)	(0.021)
Household head’s marital status				
Married b	−0.093***	−0.035	−0.061***	−0.076**
	(0.021)	(0.035)	(0.017)	(0.033)
Widowed b	−0.080***	−0.012	−0.065***	−0.080**
	(0.022)	(0.040)	(0.017)	(0.033)
Divorced b	−0.012	−0.015	−0.023	0.007
	(0.026)	(0.045)	(0.020)	(0.039)
Head’s wage (in log)	−0.003***	−0.004**	−0.001***	−0.002 *
	(0.001)	(0.001)	(0.000)	(0.001)
Other incomes (in log)	−0.004***	−0.007***	−0.006***	−0.005***
	(0.001)	(0.003)	(0.001)	(0.002)
Household asset (in log)	−0.018***	−0.013***	−0.025***	−0.020***
	(0.002)	(0.004)	(0.002)	(0.004)
Household size (equivalent)	0.000	0.016**	−0.208	−0.009 *
	(0.000)	(0.007)	(0.346)	(0.005)
Number of absent members	–0.005	0.028	–0.001	0.025**
	(0.010)	(0.020)	(0.006)	(0.011)
Observations	10,687	3,078	23,671	13,736
Ward test ($\chi^{2}$ statistics)	532.85	320.84	1,040	118.55
	[0.000]	[0.000]	[0.000]	[0.000]
Probit model goodness-of-fit test ($\chi^{2}$ statistics)	10,780	6,310	23,798	3,108
	[0.226]	[0.355]	[0.253]	[0.270]

Note: **GOVS** denotes Government support; **MHD** stands for mental health deterioration;

(a ) reference category is “having no certificate”;

(b ) reference category is “single household’s head”; Ward test ($\chi^{2}$ statistics) hypothesis is all coefficients are simultaneously equal zero; Probit model goodness-of-fit test ($\chi^{2}$ statistics) hypothesis is the model is not poor fitness; robust standard errors in parentheses; p-value of post estimation tests in squared brackets; robust standard errors in parentheses;

*** ,

** , and

*  are statistically significant at 1, 5, and 10 per cent, respectively.

## 5. Concluding remark and implications

Mental health plays an important role in achieving the overall quality of people’s health as it affects our ability to decide, form connections, and influence our world. However, issues concerning mental health, particularly in developing countries like Vietnam, have largely been ignored. Vietnam has been facing an increasing trend of mental disorders in the past two decades due to a lack of mental health literacy, discrimination, social pressure, and sub-standard mental health care. In response to this alarming trend, policy support from the Vietnamese government is provided, particularly during stressful periods such as the COVID-19 pandemic. As such, this study examines the effect of government support on mental health deterioration in Vietnam before and during the COVID-19 pandemic using recent VHLSS surveys in 2018 and 2020.

Two important and alarming findings are observed from this study. Vietnam is facing an ageing population, and young individuals experience more mental health deterioration. In addition, the income and assets of the households appear to be the buffer against mental health deterioration, implying that financial distress seems to be the main cause of mental health problems in Vietnamese households. In addition, the empirical results from our study confirm that the probability of individuals suffering mental health deterioration is positively associated with government support, implying that government support is too little. Males, as the head of the households, suffer mental health deterioration less than females. We also find that the likelihood of having mental health problems is higher for people living in urban areas. Besides, an inverted U-shaped relationship between age and mental health deterioration is observed in both the 2018 and 2020 surveys.

Several policy implications have emerged based on these insightful findings. Although fiscal policies can somehow mitigate people’s mental illness, they can never be more effective than direct mental support policies. Instead of supporting disadvantaged individuals when they are already diagnosed with mental illness, programs and campaigns to raise awareness of mental health deterioration should be a priority policy, particularly for young individuals who experience mental health problems. Doing so provides support for them to break the existing barriers to seeking help for mental illness. The Vietnamese government should also consider implementing mental health literacy and the awareness of mental health care needs. This policy can be formulated and implemented by encouraging mental health care services in educational institutions to proactively and timely prevent mental health problems for young people. Moreover, this action can create and foster a sustainable environment for psychiatrists to grow because this field is underrated in Vietnam. In addition, our findings call for financial support to disadvantaged families to ensure that these families will not fall into the mental health spectrum when they face financial distress, particularly during stressful times such as the COVID-19 pandemic.

Finally, although we were aware of an endogenous issue that could potentially affect the result by the influence of the perception of “mental health deterioration” from households, VHLSS does not carry out the question to identify the perception of mental health. Therefore, policymakers and researchers directly involved in the VHLSS can consider developing a section on mental health besides the current section on physical health in the future VHLSS. This valuable information can imply insightful meanings and recommendations for policy development to improve the health quality of Vietnamese citizens.

## Appendix 1

### Correlation matrix

**Table:** 

	MHD	GOVS	Head’s gender is male	Head’s age	The household head has no educational certificate	The household head has a pre-primary diploma	The household head has a secondary school diploma	The household head has a high school diploma	The household head has a graduate and post-graduate diploma	
**MHD**	1.000									
**GOVS**	0.094	1.000								
Head’s gender is male	−0.081	0.041	1.000							
Head’s age	0.088	−0.063	−0.163	1.000						
The household head has no educational certificate	0.103	0.172	−0.122	0.197	1.000					
The household head has a pre-primary diploma	0.040	0.067	0.010	−0.022	−0.283	1.000				
The household head has a secondary school diploma	−0.056	−0.050	0.087	−0.042	−0.311	−0.408	1.000			
The household head has a high school diploma	−0.043	−0.114	0.021	−0.071	−0.203	−0.267	−0.294	1.000		
The household head has a graduate and post-graduate diploma	−0.058	−0.112	−0.021	−0.074	−0.138	−0.181	−0.199	−0.130	1.000	
Single household	0.062	0.004	−0.159	-0.052	0.009	0.002	-0.025	0.006	0.019	
Married household	−0.112	−0.007	0.600	−0.279	−0.167	−0.021	0.078	0.061	0.053	
Widowed household	0.072	0.010	−0.530	0.371	0.184	0.014	-0.070	−0.071	−0.066	
Divorced household	0.053	−0.007	−0.173	−0.039	0.011	0.017	−0.015	−0.006	−0.010	
Household head’s wage (log)	−0.048	−0.096	0.003	−0.136	−0.108	−0.071	−0.016	0.074	0.195	
Household’s other incomes (log)	−0.087	0.077	0.185	−0.084	−0.022	0.060	0.090	−0.026	−0.184	
Household asset (log)	−0.112	−0.305	−0.002	0.120	−0.202	−0.129	0.043	0.152	0.213	
Households in urban areas	0.043	−0.217	−0.149	0.051	−0.097	−0.099	−0.063	0.133	0.224	
Household size (equivalent)	−0.006	0.006	0.009	0.027	0.004	−0.015	0.006	0.004	0.003	
Number of absent members	−0.025	−0.001	0.051	−0.070	−0.040	−0.018	0.020	0.022	0.023	
	Single household	Married household	Widowed household	Divorced household	Household head’s wage (log)	Household’s other incomes (log)	Household asset (log)	Households in urban areas	Household size (equivalent)	Number of absent members
Single household	1.000									
Married household	−0.340	1.000								
Widowed household	−0.067	−0.795	1.000							
Divorced household	−0.032	−0.386	−0.076	1.000						
Household head’s wage (log)	−0.028	0.083	−0.078	−0.011	1.000					
Household’s other incomes (log)	−0.107	0.200	−0.138	−0.081	−0.281	1.000				
Household asset (log)	−0.090	0.095	−0.038	−0.055	0.116	0.025	1.000			
Households in urban areas	0.055	−0.060	0.021	0.043	0.150	−0.204	0.321	1.000		
Household size (equivalent)	−0.036	0.019	0.004	−0.015	0.014	0.017	0.010	0.006	1.000	
Number of absent members	−0.037	0.067	−0.049	−0.021	0.049	0.067	0.022	−0.025	0.003	1.000
Note: **GOVS** denotes government support; **MHD** is mental health deterioration; ***, **, * is statistically significant at 1 per cent, 5 per cent, and 10 per cent.										

## Appendix 2

### The average marginal effect of the probit estimator of the probability that a household suffered from mental health deterioration in 2018 and 2020.

**Table:** 

The dependent variable is the probability that a household suffers from mental health	2018	2020
GOVS (in log)	0.009***	0.010***
	(0.001)	(0.001)
Head’s gender is male	−0.012**	−0.016 *
	(0.005)	(0.009)
Head’s age	0.003***	0.003**
	(0.001)	(0.002)
Square of head’s age	−0.017×10^-3^**	−0.018×10^-3^
	(0.000)	(0.000)
The household head’s education level is a		
Pre-primary diploma	−0.011**	−0.008
	(0.006)	(0.011)
Secondary school diploma	−0.029***	−0.023**
	(0.006)	(0.012)
High school diploma	−0.039***	−0.041***
	(0.007)	(0.013)
Graduate and post-graduate diploma	−0.079***	−0.078***
	(0.007)	(0.015)
Household head’s marital status b		
Married	−0.060***	−0.033
	(0.012)	(0.021)
Widowed	−0.050***	−0.021
	(0.013)	(0.023)
Divorced	−0.009	0.024
	(0.015)	(0.027)
Head’s wage (in log)	−0.002***	−0.003***
	(0.000)	(0.001)
Other incomes (in log)	−0.004***	−0.005***
	(0.000)	(0.002)
Household asset (in log)	−0.023***	−0.018***
	(0.002)	(0.003)
Households in urban areas	0.047***	0.042***
	(0.004)	(0.008)
Household size (equivalent)	−0.268	0.000
	(0.322)	(0.004)
Number of absent members	0.001	0.027***
	(0.005)	(0.010)
Observations	34,358	9,364
Provincial fixed effect	YES	YES
Ward test ( $\chi^{2}$ statistics)	2,283	708
	[0.000]	[0.000]
Probit model goodness-of-fit test ( $\chi^{2}$ statistics)	34,385	9,372
	[0.339]	[0.258]
Note: **GOVS** is Government supports; **MHD** stands for mental health deterioration; (a) reference category has no certificate; (b) reference category is single household’s head; Ward test ( $\chi^{2}$ statistics) hypothesis is all coefficients are simultaneously equal zero; Probit model goodness-of-fit test ( $\chi^{2}$ statistics) hypothesis is the model is not poor fitness; robust standard errors in parentheses; p-value of post estimation tests in squared brackets; ***, **, and * are statistically significant at 1, 5, and 10 per cent, respectively.		

## Appendix 3

### The average marginal effect of the probit estimator on the probability that a household suffers from mental health deterioration: the non-poor versus poor households

**Table:** 

The dependent variable is the probability that a household suffers from MHD.	Non-poor households		Poor households	
	*2018*	*2020*	*2018*	*2020*
GOVS (in log)	0.005***	0.006***	0.005	0.014**
	(0.001)	(0.001)	(0.005)	(0.007)
Head’s gender is male	−0.010**	−0.020**	−0.025	0.062
	(0.005)	(0.009)	(0.025)	(0.049)
Head’s age	0.003***	0.003**	0.010***	0.005
	(0.001)	(0.002)	(0.003)	(0.006)
Square of head’s age	−0.016×10^-3^**	−0.016×10^-3^	−0.073×10^-3^**	−0.042×10^-3^
	(0.000)	(0.000)	(0.000)	(0.000)
Household head’s education level				
Pre-primary diploma a	−0.014**	−0.003	0.040 *	−0.051
	(0.006)	(0.011)	(0.021)	(0.039)
Secondary school diploma a	−0.028***	−0.021 *	−0.011	0.011
	(0.006)	(0.012)	(0.023)	(0.051)
High school diploma, a	−0.038***	−0.037***	0.037	−0.027
	(0.007)	(0.013)	(0.037)	(0.080)
Graduate and post-graduate diploma a	−0.073***	−0.074***	−0.159 *	0.016
	(0.007)	(0.014)	(0.089)	(0.182)
Household head’s marital status				
Married b	−0.050***	−0.037	−0.052	0.026
	(0.012)	(0.023)	(0.042)	(0.067)
Widowed b	−0.039***	−0.038	−0.091**	0.157**
	(0.013)	(0.024)	(0.042)	(0.073)
Divorced b	−0.003	−0.000	−0.025	0.244***
	(0.015)	(0.028)	(0.052)	(0.093)
Head’s wage (in log)	−0.002***	−0.002***	−0.003	−0.007 *
	(0.000)	(0.001)	(0.002)	(0.004)
Other incomes (in log)	−0.003***	−0.005***	−0.011***	−0.005
	(0.000)	(0.001)	(0.002)	(0.013)
Household asset (in log)	−0.021***	−0.015***	0.003	−0.029 *
	(0.002)	(0.003)	(0.008)	(0.015)
Households in urban areas	0.041***	0.043***	0.135***	0.008
	(0.004)	(0.008)	(0.028)	(0.051)
Household size (equivalent)	−0.302	0.002	0.000	−0.001
	(0.309)	(0.004)	(0.000)	(0.021)
Number of absent members	0.001	0.026***	0.008	−0.032
	(0.005)	(0.010)	(0.025)	(0.056)
Observations	31,912	8,659	2,446	684
Provincial fixed effect	YES	YES	YES	YES
Ward test ( $\chi^{2}$ statistics)	1,777	575	397	129
	[0.000]	[0.000]	[0.000]	[0.000]
Probit model goodness-of-fit test ( $\chi^{2}$ statistics)	32,071	8,540	2,462	712
	[0.171]	[0.615]	[0.084]	[0.003]
Note: **GOVS** is Government supports; **MHD** stands for mental health deterioration; (a) reference category has no certificate; (b) reference category is single household’s head; Ward test ( $\chi^{2}$ statistics) hypothesis is all coefficients are simultaneously equal zero; Probit model goodness-of-fit test ( $\chi^{2}$ statistics) hypothesis is the model is not poor fitness; robust standard errors in parentheses; p-value of post estimation tests in squared brackets; robust standard errors in parentheses; ***, **, and * are statistically significant at 1, 5, and 10 per cent, respectively.				

## Appendix 4

### The average marginal effect of the probit estimator on the probability that a household suffers from mental health deterioration: Female-headed households (FHHs) versus Male-headed households (MHHs)

**Table:** 

The dependent variable is the probability that a household suffers from MHD.	*Sub-sample of FHHs*		*Sub-sample of MHHs*	
	*2018*	*2020*	*2018*	*2020*
GOVS (in log)	0.014***	0.013***	0.008***	0.009***
	(0.001)	(0.003)	(0.001)	(0.001)
Head’s age	0.007***	0.009***	0.002 *	0.001
	(0.002)	(0.003)	(0.001)	(0.002)
Square of head’s age	−0.048×10^-3^***	−0.069×10^-3^***	−0.002×10^-3^	0.010×10^-3^
	(0.000)	(0.000)	(0.000)	(0.000)
Household head’s education level				
Pre-primary diploma	−0.014	0.002	−0.012 *	−0.013
	(0.012)	(0.024)	(0.006)	(0.012)
Secondary school diploma a	−0.025 *	−0.037	−0.031***	−0.023 *
	(0.013)	(0.026)	(0.007)	(0.013)
High school diploma, a	−0.047***	−0.069**	−0.038***	−0.034**
	(0.015)	(0.028)	(0.008)	(0.014)
Graduate and post-graduate diploma a	−0.091***	−0.085**	−0.077***	−0.079***
	(0.016)	(0.035)	(0.008)	(0.016)
Household head’s marital status				
Married b	−0.072***	−0.047	−0.040**	−0.002
	(0.018)	(0.034)	(0.019)	(0.027)
Widowed b	−0.064***	−0.038	−0.035	0.004
	(0.018)	(0.035)	(0.022)	(0.035)
Divorced b	−0.054***	−0.045	0.058**	0.136***
	(0.020)	(0.039)	(0.026)	(0.044)
Head’s wage (in log)	−0.005***	−0.005***	−0.001***	−0.002***
	(0.001)	(0.002)	(0.000)	(0.001)
Other incomes (in log)	−0.004***	−0.009***	−0.004***	−0.004**
	(0.001)	(0.003)	(0.000)	(0.002)
Household asset (in log)	−0.025***	−0.027***	−0.022***	−0.014***
	(0.003)	(0.006)	(0.002)	(0.003)
Households in urban areas	0.062***	0.022	0.041***	0.049***
	(0.010)	(0.018)	(0.005)	(0.009)
Household size (equivalent)	−0.667 *	0.004	0.000	0.001
	(0.399)	(0.009)	(0.000)	(0.004)
Number of absent members	−0.008	0.011	0.003	0.031***
	(0.013)	(0.027)	(0.005)	(0.010)
Observations	8,671	2,354	25,686	6,913
Provincial fixed effect	YES	YES	YES	YES
Ward test ( $\chi^{2}$ statistics)	652	232	1,558	514
	[0.000]	[0.000]	[0.000]	[0.000]
Probit model goodness-of-fit test ( $\chi^{2}$ statistics)	8750	2,291	25,661	6,904
	[0.114]	[0.428]	[0.406]	[0.275]
Note: **GOVS** is Government supports; **MHD** stands for mental health deterioration; (a) reference category has no certificate; (b) reference category is single household’s head; Ward test ( $\chi^{2}$ statistics) hypothesis is all coefficients are simultaneously equal zero; Probit model goodness-of-fit test ( $\chi^{2}$ statistics) hypothesis is the model is not poor fitness; robust standard errors in parentheses; p-value of post estimation tests in squared brackets; robust standard errors in parentheses; ***, **, and * are statistically significant at 1, 5, and 10 per cent, respectively.				

## Appendix 5

### The average marginal effect of the probit estimator on the probability that a household suffers from mental health deterioration: Rural area versus Urban area

**Table:** 

The dependent variable is the probability that a household suffers from MHD.	*Sub-sample of urban area*		*Sub-sample of rural area*	
	*2018*	*2020*	*2018*	*2020*
GOVS (in log)	0.008***	0.011***	0.013***	0.006**
	(0.001)	(0.001)	(0.001)	(0.003)
Head’s gender is male	−0.015**	−0.023 *	−0.005	0.006
	(0.006)	(0.012)	(0.008)	(0.015)
Head’s age	0.003***	0.001	0.005***	0.010***
	(0.001)	(0.002)	(0.002)	(0.003)
Square of head’s age	−0.013×10^-3^	0.005×10^-3^	−0.032×10^-3^**	−0.068×10^-3^**
	(0.000)	(0.000)	(0.000)	(0.000)
Household head’s education level				
Pre-primary diploma a	−0.006	−0.006	−0.022 *	−0.007
	(0.006)	(0.012)	(0.013)	(0.025)
Secondary school diploma a	−0.019***	−0.027**	−0.051***	−0.004
	(0.006)	(0.013)	(0.013)	(0.026)
High school diploma, a	−0.034***	−0.043***	−0.053***	−0.035
	(0.008)	(0.015)	(0.014)	(0.026)
Graduate and post-graduate diploma a	−0.079***	−0.062***	−0.095***	−0.093***
	(0.009)	(0.022)	(0.014)	(0.026)
Household head’s marital status				
Married b	−0.053***	−0.061**	−0.074***	−0.020
	(0.016)	(0.030)	(0.020)	(0.034)
Widowed b	−0.052***	−0.058 *	−0.053**	0.004
	(0.016)	(0.031)	(0.021)	(0.039)
Divorced b	−0.025	0.020	−0.002	−0.003
	(0.019)	(0.037)	(0.025)	(0.044)
Head’s wage (in log)	−0.002***	−0.003***	−0.003***	−0.004**
	(0.000)	(0.001)	(0.001)	(0.002)
Other incomes (in log)	−0.005***	−0.004**	−0.003***	−0.006**
	(0.001)	(0.002)	(0.001)	(0.003)
Household asset (in log)	−0.028***	−0.025***	−0.022***	−0.017***
	(0.002)	(0.004)	(0.002)	(0.005)
Household size (equivalent)	−0.227	−0.004	0.000	0.012 *
	(0.293)	(0.005)	(0.000)	(0.007)
Number of absent members	–0.001	0.024**	0.004	0.035 *
	(0.005)	(0.011)	(0.010)	(0.020)
Observations	23,671	6,271	10,687	2,895
Provincial fixed effect	YES	YES	YES	YES
Ward test ( $\chi^{2}$ statistics)	1,647	564	806	242
	[0.000]	[0.000]	[0.000]	[0.000]
Probit model goodness-of-fit test ( $\chi^{2}$ statistics)	23,422	6,127	10,878	2,867
	[0.782]	[0.722]	[0.032]	[0.275]
Note: **GOVS** denotes Government support; **MHD** stands for mental health deterioration; (a) reference category is “having no certificate”; (b) reference category is “single household’s head”; Ward test ( $\chi^{2}$ statistics) hypothesis is all coefficients are simultaneously equal zero; Probit model goodness-of-fit test ( $\chi^{2}$ statistics) hypothesis is the model is not poor fitness; robust standard errors in parentheses; p-value of post estimation tests in squared brackets; robust standard errors in parentheses; ***, **, and * are statistically significant at 1, 5, and 10 per cent, respectively.				

## Appendix 6

### The average marginal effect of the logit estimator of the probability that a household suffered from mental health deterioration in 2018 and 2020

**Table:** 

The dependent variable is the probability that a household suffers from mental health	2018	2020
GOVS (in log)	0.007***	0.008***
	(0.001)	(0.001)
Head’s gender is male	−0.016***	−0.024**
	(0.005)	(0.010)
Head’s age	0.005***	0.005***
	(0.001)	(0.002)
Square of head’s age	−0.027×10^-3^***	−0.029×10^-3^***
	(0.000)	(0.000)
The household head’s education level is a		
Pre-primary diploma	−0.016***	−0.010
	(0.006)	(0.011)
Secondary school diploma	−0.054***	−0.044***
	(0.006)	(0.011)
High school diploma	−0.060***	−0.058***
	(0.007)	(0.012)
Graduate and post-graduate diploma	−0.098***	−0.093***
	(0.007)	(0.014)
Household head’s marital status b		
Married	−0.071***	−0.046**
	(0.013)	(0.023)
Widowed	−0.068***	−0.041 *
	(0.013)	(0.024)
Divorced	−0.014	0.014
	(0.015)	(0.028)
Head’s wage (in log)	−0.002***	−0.002***
	(0.000)	(0.001)
Other incomes (in log)	−0.005***	−0.006***
	(0.000)	(0.002)
Household asset (in log)	−0.020***	−0.014***
	(0.001)	(0.003)
Households in urban areas	0.059***	0.050***
	(0.004)	(0.008)
Household size (equivalent)	−0.266	−0.001
	(0.325)	(0.004)
Number of absent members	−0.002	0.025***
	(0.005)	(0.010)
Observations	34,358	9,364
Ward test ( $\chi^{2}$ statistics)	1,621	412
	[0.000]	[0.000]
Logit model goodness-of-fit test ( $\chi^{2}$ statistics)	34,385	9,336
	[0.431]	[0.526]
Note: **GOVS** is Government supports; **MHD** stands for mental health deterioration; (a) reference category has no certificate; (b) reference category is single household’s head; Ward test ( $\chi^{2}$ statistics) hypothesis is all coefficients are simultaneously equal zero; Logit model goodness-of-fit test ( $\chi^{2}$ statistics) hypothesis is the model is not poor fitness; robust standard errors in parentheses; p-value of post estimation tests in squared brackets; ***, **, and * are statistically significant at 1, 5, and 10 per cent, respectively.		

## Appendix 7

### The average marginal effect of the logit estimator on the probability that a household suffers from mental health deterioration: the non-poor versus poor households

**Table:** 

The dependent variable is the probability that a household suffers from MHD.	Non-poor households		Poor households	
	*2018*	*2020*	*2018*	*2020*
GOVS (in log)	0.004***	0.005***	0.010 *	0.016**
	(0.001)	(0.001)	(0.005)	(0.007)
Head’s gender is male	−0.013**	−0.027***	−0.039	0.023
	(0.005)	(0.010)	(0.027)	(0.049)
Head’s age	0.004***	0.004***	0.015***	0.014**
	(0.001)	(0.002)	(0.003)	(0.006)
Square of head’s age	−0.024×10^-3^s	−0.024×10^-3^ *	−0.102×10^-3^***	−0.100×10^-3^ *
	(0.000)	(0.000)	(0.000)	(0.000)
Household head’s education level				
Pre-primary diploma a	−0.020***	−0.004	0.035	−0.049
	(0.006)	(0.012)	(0.022)	(0.041)
Secondary school diploma a	−0.055***	−0.043***	−0.025	−0.033
	(0.006)	(0.011)	(0.023)	(0.048)
High school diploma, a	−0.061***	−0.056***	0.038	−0.010
	(0.007)	(0.012)	(0.038)	(0.080)
Graduate and post-graduate diploma a	−0.092***	−0.089***	−0.101	0.242
	(0.007)	(0.014)	(0.158)	(0.283)
Household head’s marital status				
Married b	−0.061***	−0.050**	−0.086 *	0.002
	(0.013)	(0.025)	(0.046)	(0.072)
Widowed b	−0.058***	−0.057**	−0.124***	0.073
	(0.013)	(0.026)	(0.044)	(0.074)
Divorced b	−0.009	−0.011	−0.027	0.204**
	(0.016)	(0.030)	(0.057)	(0.096)
Head’s wage (in log)	−0.002***	−0.002***	0.002	0.001
	(0.000)	(0.001)	(0.002)	(0.004)
Other incomes (in log)	−0.004***	−0.006***	−0.017***	−0.003
	(0.000)	(0.001)	(0.002)	(0.013)
Household asset (in log)	−0.019***	−0.012***	0.006	−0.018
	(0.002)	(0.003)	(0.008)	(0.016)
Households in urban areas	0.055***	0.049***	0.155***	0.059
	(0.004)	(0.008)	(0.027)	(0.051)
Household size (equivalent)	−0.259	0.002	0.000	−0.046**
	(0.306)	(0.004)	(0.000)	(0.021)
Number of absent members	−0.001	0.024**	−0.001	0.026
	(0.005)	(0.010)	(0.027)	(0.049)
Observations	31,912	8,659	2,446	705
Ward test ( $\chi^{2}$ statistics)	228	295	1,204	47.3
	[0.000]	[0.000]	[0.000]	[0.000]
Logit model goodness-of-fit test ( $\chi^{2}$ statistics)	31,850	8,629	2,420	706
	[0.567]	[0.534]	[0.546]	[0.301]
Note: **GOVS** is Government supports; **MHD** stands for mental health deterioration; (a) reference category has no certificate; (b) reference category is single household’s head; Ward test ( $\chi^{2}$ statistics) hypothesis is all coefficients are simultaneously equal zero; Logit model goodness-of-fit test ( $\chi^{2}$ statistics) hypothesis is the model is not poor fitness; robust standard errors in parentheses; p-value of post estimation tests in squared brackets; robust standard errors in parentheses; ***, **, and * are statistically significant at 1, 5, and 10 per cent, respectively.				

## Appendix 8

### The average marginal effect of the logit estimator on the probability that a household suffers from mental health deterioration: Female-headed households (FHHs) versus Male-headed households (MHHs)

**Table:** 

The dependent variable is the probability that a household suffers from MHD.	*Sub-sample of FHHs*		*Sub-sample of MHHs*	
	*2018*	*2020*	*2018*	*2020*
GOVS (in log)	0.012***	0.009***	0.005***	0.008***
	(0.001)	(0.002)	(0.001)	(0.001)
Head’s age	0.007***	0.009***	0.003***	0.003
	(0.002)	(0.003)	(0.001)	(0.002)
Square of head’s age	−0.050×10^-3^***	−0.061×10^-3^**	−0.015×10^-3^ *	−0.006×10^-3^
	(0.000)	(0.000)	(0.000)	(0.000)
Household head’s education level				
Pre-primary diploma	−0.024**	−0.010	−0.015**	−0.011
	(0.012)	(0.022)	(0.007)	(0.013)
Secondary school diploma a	−0.057***	−0.053**	−0.053***	−0.042***
	(0.013)	(0.023)	(0.007)	(0.012)
High school diploma, a	−0.072***	−0.080***	−0.057***	−0.051***
	(0.015)	(0.026)	(0.007)	(0.014)
Graduate and post-graduate diploma a	−0.118***	−0.108***	−0.093***	−0.091***
	(0.016)	(0.032)	(0.008)	(0.015)
Household head’s marital status				
Married b	−0.071***	−0.053	−0.067***	−0.024
	(0.018)	(0.036)	(0.022)	(0.032)
Widowed b	−0.073***	−0.059 *	−0.062***	−0.017
	(0.018)	(0.036)	(0.024)	(0.038)
Divorced b	−0.053**	−0.051	0.039	0.115**
	(0.021)	(0.040)	(0.028)	(0.048)
Head’s wage (in log)	−0.005***	−0.004**	−0.001***	−0.002**
	(0.001)	(0.002)	(0.000)	(0.001)
Other incomes (in log)	−0.005***	−0.009***	−0.005***	−0.005***
	(0.001)	(0.003)	(0.000)	(0.002)
Household asset (in log)	−0.021***	−0.021***	−0.020***	−0.011***
	(0.003)	(0.006)	(0.002)	(0.003)
Households in urban areas	0.073***	0.031 *	0.053***	0.059***
	(0.009)	(0.017)	(0.005)	(0.009)
Household size (equivalent)	–0.665 *	0.002	0.000	–0.001
	(0.403)	(0.009)	(0.000)	(0.004)
Number of absent members	–0.015	–0.000	0.002	0.031***
	(0.014)	(0.027)	(0.005)	(0.010)
Observations	8,671	2,451	25,686	6,913
Ward test ( $\chi^{2}$ statistics)	454	117	1,048	265
	[0.000]	[0.000]	[0.000]	[0.000]
Logit model goodness-of-fit test ( $\chi^{2}$ statistics)	8,719	2,459	25,731	6,861
	[0.309]	[0.358]	[0.393]	[0.617]
Note: **GOVS** is Government supports; **MHD** stands for mental health deterioration; (a) reference category has no certificate; (b) reference category is single household’s head; Ward test ( $\chi^{2}$ statistics) hypothesis is all coefficients are simultaneously equal zero; Logit model goodness-of-fit test ( $\chi^{2}$ statistics) hypothesis is the model is not poor fitness; robust standard errors in parentheses; p-value of post estimation tests in squared brackets; robust standard errors in parentheses; ***, **, and * are statistically significant at 1, 5, and 10 per cent, respectively.				

## Appendix 9

### The average marginal effect of the logit estimator on the probability that a household suffers from mental health deterioration: Rural area versus Urban area

**Table:** 

The dependent variable is the probability that a household suffers from MHD.	*Sub-sample of urban area*		*Sub-sample of rural area*	
	*2018*	*2020*	*2018*	*2020*
GOVS (in log)	0.010***	0.008***	0.006***	0.007***
	(0.001)	(0.002)	(0.001)	(0.001)
Head’s gender is male	–0.010	–0.010	–0.020***	–0.030**
	(0.008)	(0.015)	(0.007)	(0.013)
Head’s age	0.006***	0.012***	0.004***	0.002
	(0.002)	(0.003)	(0.001)	(0.002)
Square of head’s age	–0.036×10^-3^**	–0.086×10^-3^***	–0.026×10^-3^***	–0.001×10^-3^
	(0.000)	(0.000)	(0.000)	(0.000)
Household head’s education level				
Pre-primary diploma a	–0.024 *	0.000	–0.013**	–0.015
	(0.013)	(0.022)	(0.006)	(0.012)
Secondary school diploma a	–0.067***	–0.022	–0.045***	–0.053***
	(0.013)	(0.022)	(0.006)	(0.012)
High school diploma, a	–0.064***	–0.045**	–0.058***	–0.066***
	(0.013)	(0.022)	(0.008)	(0.015)
Graduate and post-graduate diploma a	–0.107***	–0.092***	–0.099***	–0.086***
	(0.014)	(0.023)	(0.009)	(0.022)
Household head’s marital status				
Married b	–0.091***	–0.033	–0.057***	–0.071**
	(0.021)	(0.036)	(0.016)	(0.032)
Widowed b	–0.079***	–0.015	–0.061***	–0.075**
	(0.022)	(0.040)	(0.016)	(0.032)
Divorced b	–0.012	–0.018	–0.021	0.008
	(0.026)	(0.045)	(0.019)	(0.038)
Head’s wage (in log)	–0.003***	–0.004***	–0.001***	–0.001
	(0.001)	(0.001)	(0.000)	(0.001)
Other incomes (in log)	–0.004***	–0.007***	–0.006***	–0.005***
	(0.001)	(0.003)	(0.001)	(0.002)
Household asset (in log)	–0.018***	–0.014***	–0.024***	–0.020***
	(0.002)	(0.005)	(0.002)	(0.004)
Household size (equivalent)	0.000	0.014**	–0.213	–0.010**
	(0.000)	(0.007)	(0.311)	(0.005)
Number of absent members	–0.006	0.028	–0.002	0.025**
	(0.011)	(0.020)	(0.006)	(0.011)
Observations	10,687	3,078	23,671	6,286
Ward test ( $\chi^{2}$ statistics)	543	116	1,043	328
	[0.000]	[0.000]	[0.000]	[0.000]
Logit model goodness-of-fit test ( $\chi^{2}$ statistics)	10,758	3,108	23,654	6,273
	[0.275]	[0.273]	[0.497]	[0.482]
Note: **GOVS** denotes Government support; **MHD** stands for mental health deterioration; (a) reference category is “having no certificate”; (b) reference category is “single household’s head”; Ward test ( $\chi^{2}$ statistics) hypothesis is all coefficients are simultaneously equal zero; Logit model goodness-of-fit test ( $\chi^{2}$ statistics) hypothesis is the model is not poor fitness; robust standard errors in parentheses; p-value of post estimation tests in squared brackets; robust standard errors in parentheses; ***, **, and * are statistically significant at 1, 5, and 10 per cent, respectively.				
